# Biases in clinical trials with sequential monitoring

**DOI:** 10.1186/1745-6215-12-S1-A50

**Published:** 2011-12-13

**Authors:** Manjula Schou, Ian C Marschner

**Affiliations:** 1 Department of Statistics, Macquarie University, Sydney, NSW 2109, Australia

## Objectives

It is well known that a sequentially monitored clinical trial that stops early for benefit has a crude treatment difference that overestimates the true treatment effect. This has led to extended debate in the literature, with some researchers arguing that early stopping is an important source of bias in meta-analyses of clinical trials. We therefore investigated the implications of excluding studies that stopped early, so-called truncated studies, from estimation of treatment effects.

## Methods

The effect of excluding truncated studies was investigated by examining the statistical properties of sequentially monitored studies conditional on reaching the planned final analysis. Using theory and simulation we studied clinical trials with standard sequential rules for stopping early due to benefit. As well as estimation bias, we studied information bias measured as the difference between standard measures of the statistical information, such as sample size, and the actual information based on the conditional sampling distribution.

## Results

We found exclusion of truncated studies leads to both estimation bias and information bias. Treatment differences are underestimated and information is overestimated. Most importantly, the magnitude of information bias is an increasing function of the magnitude of estimation bias. This has important implications for meta-analyses that typically weight by sample size. In particular, it means that studies with the most biased treatment effect are the most overweighted studies in a meta-analysis. The magnitudes of both estimation and information biases can be practically significant. When all studies were included in meta-analyses, both truncated and non-truncated, the estimation of treatment effects was unbiased.

## Conclusions

Crude methods of analysis for sequentially monitored studies can lead to underestimation bias if truncated studies are excluded from estimation of treatment effects. Furthermore, information bias resulting from this exclusion leads to a double whammy effect, in which the most biased studies are the most overweighted studies in a meta-analysis. Since exclusion of truncated studies is problematic, we advocate wider reporting of adjusted estimates of treatment effects that take account of any interim monitoring, and recommend that all studies, both truncated and non-truncated, are included in meta-analyses.

